# Unisexual Reproduction Drives Evolution of Eukaryotic Microbial Pathogens

**DOI:** 10.1371/journal.ppat.1003674

**Published:** 2013-10-31

**Authors:** Marianna Feretzaki, Joseph Heitman

**Affiliations:** 1 Department of Molecular Genetics and Microbiology, Duke University Medical Center, Durham, North Carolina, United States of America; 2 Department of Medicine, Duke University Medical Center, Durham, North Carolina, United States of America; 3 Department of Pharmacology and Cancer Biology, Duke University Medical Center, Durham, North Carolina, United States of America; University of Wisconsin Medical School, United States of America

## Introduction

**Figure 1:**
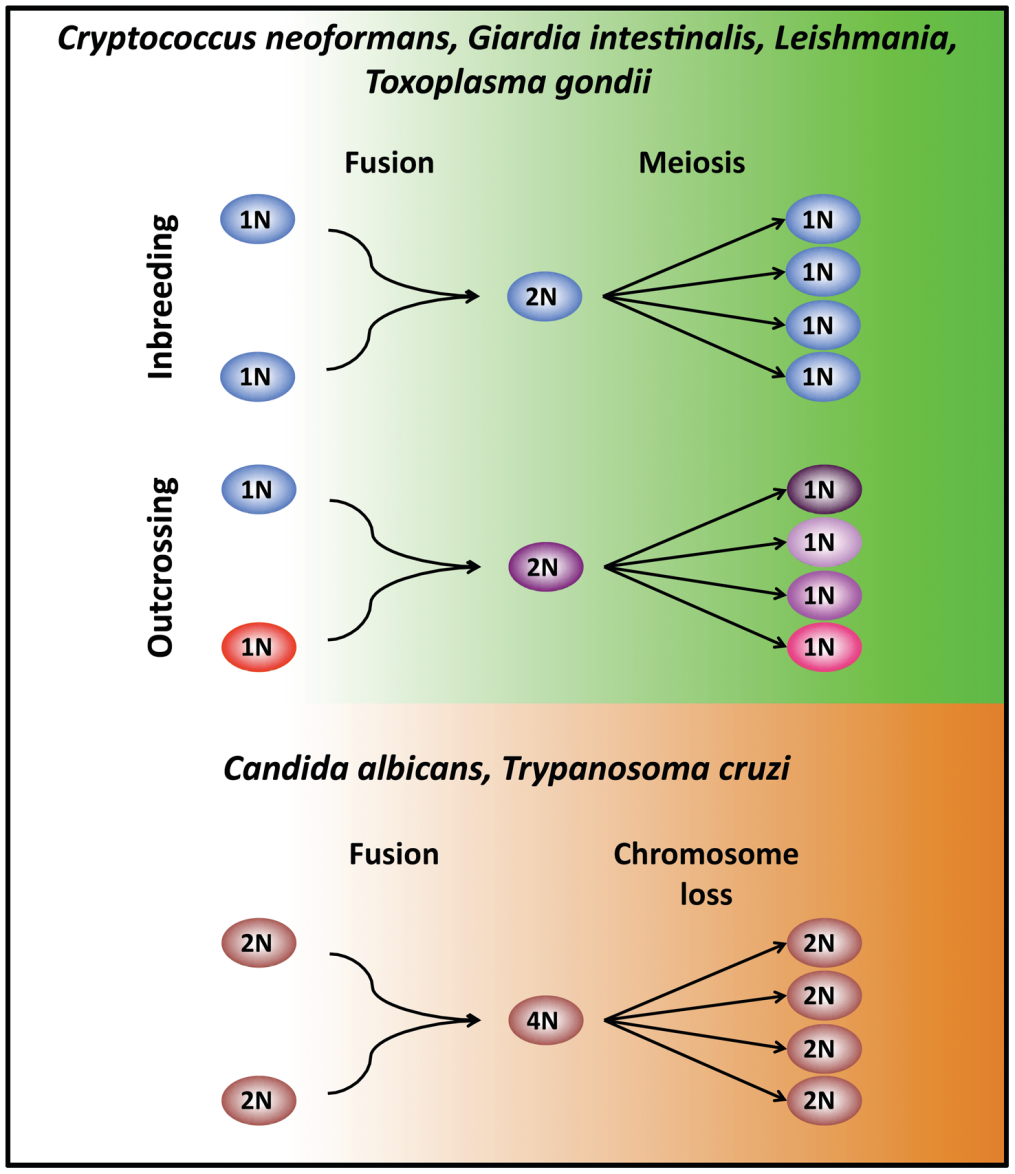
Modes of unisexual reproduction. In haploid eukaryotes, cells (1N) of the same mating type can fuse or undergo endoreplication to generate a diploid intermediate (2N). DNA replication without cell division follows and meiosis produces four recombinant progeny (1N). Unisexual reproduction can also occur in 1) a clonal population, promoting inbreeding, or 2) between cells of the same mating type but of distinct genetic lineages to enable outcrossing. A similar unisexual cycle is observed in *Candida* and possibly also *Trypanosoma*, where diploid cells (2N) fuse to generate a tetraploid intermediate (4N). The resulting tetraploid cells (4N) return to the diploid state (2N) through either parasexual chromosome loss that occurs stochastically independent of meiosis (*C. albicans*, and possibly *T. cruzi*) or sexually via meiosis (*T. brucei*).

Genetic exchange occurs via horizontal gene transfer in bacteria and archea or sexual reproduction in fungal and parasitic eukaryotic microbes. Sexual reproduction is universal, or nearly so, in eukaryotes. Until recently, most eukaryotic microbial pathogens were thought to be clonal and asexual due to the absence of a compatible partner or the lack of morphological or population genetic evidence for sexual reproduction [1]. However, many of these eukaryotic pathogens have been found recently to have extant cryptic sexual cycles (Figure 1). Sex enables microbial pathogens to reshuffle their genomes, increase genetic diversity, purge deleterious mutations, and produce infectious propagules.

## Unisexual Reproduction in Fungi

Sexual reproduction involving cells of opposite mating types or sexes comes with costs. Locating a compatible partner and undergoing mating and meiosis requires time and energy. In addition, sexual reproduction introduces genetic diversity, but in so doing rearranges well-adapted genomic configurations. In contrast, sexual reproduction involving cells of only one type (unisexual reproduction), via either mother-daughter cell-cell fusion or endoreplication, lowers the barrier to locating a compatible mating partner. Unisexual reproduction ameliorates the cost of losing a well-adapted phenotype in a particular niche while introducing more limited genetic diversity that may enhance the fitness of progeny in response to environmental changes, including drug treatments.

*Cryptococcus neoformans* is a basidiomycetous pathogenic yeast found in both the environment and infected hosts. *Cryptococcus* has a bipolar mating system with two mating types, **a** and α [2], and a well-defined sexual cycle involving cells of opposite mating type that fuse and undergo a dimorphic yeast-hyphal transition. At the hyphal tips, basidia form where meiosis occurs followed by multiple rounds of mitosis, producing long spore chains [3], [4]. Spores are infectious propagules that are inhaled by the host [5], [6].

Although *C. neoformans* has a defined **a**-α sexual cycle, the α mating type predominates in environmental and clinical isolates, and in many niches the population is exclusively α, dramatically limiting opportunities for **a**-α sexual reproduction [7]. Recent studies have shown that *C. neoformans* undergoes unisexual reproduction that involves an alternative dimorphic transition to hyphal growth, production of basidia, meiosis, and sporulation [5], [6], [8]. This commonly occurs with α strains but has also been reported to occur with some strains of the **a** mating type [3], [9], [10]. The pathway is controlled by multiple genetic loci as a quantitative trait, of which the *MAT*α locus allele provides the major contribution [10]. Diploidization occurs during unisexual reproduction: either early to produce a diploid yeast that then produces a diploid monokaryotic hyphae, or late in which a haploid yeast produces a haploid monokaryotic hyphae in which karyogamy is delayed until the basidia form (similar to **a**-α sexual reproduction) [10], [11].

Meiotic recombination occurs at a similar frequency in spores produced by either opposite sexual or unisexual reproduction [8]. Moreover, the key meiotic genes *SPO11* and *DMC1* (which induce and repair DNA DSBs that provoke meiotic recombination) are both dispensable for hyphal and basidia development, but critical for sporulation during unisexual reproduction: the *dmc1* and *spo11* mutants produce fewer spores, often in only two instead of four chains, and their germination frequency is severely impaired resulting in only 2% or 3.7% the wild-type level of viable spore production in these meiotic mutants [8], [12]. Thus, unisexual reproduction is a complete sexual cycle involving meiotic production of spores.

While the unisexual cycle has been directly observed only under laboratory conditions, mounting population genetic evidence supports that unisexual cycles occur in nature in both clonal and genetically divergent populations leading to recombination in exclusively α populations and also resulting in diploid intermediates or products with αADα or αAAα genotypes [13]–[19]. Moreover, recent studies have shown that unisexual reproduction between genetically identical cells generates phenotypic and genotypic diversity, including SNPs, chromosomal translocations, and aneuploidy, which may enhance competitive fitness in different environments [20]. Interestingly, population genetic analyses also implicate unisexual reproduction in the origins of the strains responsible for the outbreak caused by the sibling species *Cryptococcus gattii* on Vancouver Island and in the Pacific Northwest. Unisex may also be producing the spores causing the outbreak given that the lineages responsible are all of the α mating type [15].

These studies illustrate how unisexual reproduction may 1) admix genetic diversity to facilitate adaptive selection and 2) serve as a mutagen to generate genetic diversity *de novo* in otherwise clonal populations. In the latter case, the intermediate diploid state could also serve as a capacitor for evolution, allowing the accumulation of multiple recessive mutations whose combination may be advantageous when released into the haploid state, as has been observed in *A. nidulans* [21].

*Candida albicans*, the most common human fungal pathogen, was until recently thought to be strictly asexual. An extant heterothallic parasexual cycle has been defined involving diploid **a**/**a** and α/α cells that undergo cell-cell fusion and nuclear fusion, yielding a tetraploid intermediate [22]–[24]. Surprisingly, meiosis has not yet been observed in *C. albicans*, and the tetraploid returns to the diploid state through stochastic parasexual chromosome loss, which generates considerable aneuploidy, as well as infrequent Spo11-dependent recombination [25]. Unexpectedly, **a/a** cells can express both the **a** and α pheromone genes in response to nutrient limitation, which led to the discovery of a unisexual cycle in *C. albicans* [26]. Mutation of Bar1, a protease that cleaves α-factor to regulate autocrine and paracrine signaling, enables an **a**-**a** unisexual cycle [27]. This homothallic parasexual cycle is also induced in ménage à trois matings in which a third mating type partner in limited abundance donates pheromone to promote **a**-**a** or α-α mating [27]. Remarkably, pheromones from other species such as *Candida dubliniensis* or *Candida parapsilosis* can induce unisexual reproduction of *C. albicans* [28].

Similar to *C. neoformans*, where unisexual reproduction can introduce genomic changes in a clonal population [20], the aneuploidy-prone parasexual cycle also induces a range of novel genotypes in *C. albicans* that may provide selective advantages in novel environments. Although aneuploidy can be deleterious, in fungi aneuploidy can also confer drug resistance to antifungal therapy and promote pathogen survival in the host [29]–[31]. Moreover, parasexual reproduction in *C. albicans* is linked to a morphogenic change from white to opaque cells. Opaque cells are specialized in mating, while white cells are highly virulent. Although white cells are infertile, pheromone from rare opaque cells stimulates adhesion and biofilm formation of white cells [32]. Thus, roles of unisexual development may extend beyond mating and genetic diversity with the capacity to induce virulence attributes in hostile environments.

There are other pathogenic fungi once thought to be asexual that we now appreciate may be cryptically sexual or unisexual. Recent studies on the AIDS-associated pathogen *Penicillium marneffei* provide genetic evidence of a sexual cycle, and the presence of a clonal population with limited recombination rates suggests selfing may also occur in this fungus [33]. Unisexual reproduction is so far uncommon in the fungal kingdom, but many different species undergo other forms of homothallic sexual cycles. Some fungi harbor both mating type locus alleles or idiomorphs in their genome, which can be linked or unlinked, while others can switch mating type (*Saccharomyces cerevisiae* and *Schizosaccharomyces pombe* and related yeasts). Others have a single mating type locus and complete a sexual cycle in the absence of an opposite–mating type partner. Others species, such as *N. africana*, *N. galapagosensis*, *N. dodgei*, and *N. lineolata*, may undergo unisexual reproduction given that their populations appear to harbor one *MAT* locus idiomorph yet they are sexual [34]–[38]. Furthermore, the obligate intracellular microsporidian fungal pathogen *Encephalitozoon cuniculi* contains two HMG domains similar to the *MAT* locus of zygomycetes, although an extant sexual cycle has not been observed [39]. Recent studies have revealed low levels of heterozygosity in four *E. cuniculi* strains, indicative of a diploid nuclear state that may reflect a cryptic unisexual cycle in this “asexual” fungus [40].

## Unisexual Reproduction in Parasites

Among a broader group of microbial pathogens, the protozoan parasites also harbor extant sexual cycles that in some cases are now known to be cryptic or unisexual. Although these pathogens exhibit interesting and unusual sexual cycles, essentially nothing is as yet known about how sexes or mating types are established.

Until recently the intestinal parasite *Giardia intestinalis* was thought to be asexual; however, population genetics studies revealed evidence of genetic exchange, and the genome harbors a suite of meiotic genes [41], [42]. This diplomonad parasite is binucleate, and the two nuclei of a single isolate can fuse to exchange genetic information, followed by homologous recombination that is possibly directed by meiotic gene homologs [43]. The cues that trigger cell-cell fusion in the population remain to be explored. However, high levels of genetic exchange in the population provide evidence that outcrossing is likely occurring.

In the pathogen *Leishmania*, which is highly clonal and was therefore thought to be asexual, recent studies have revealed that an extant sexual cycle occurs in the sand fly vector [44]–[46]. Both outcrossing and selfing have been observed in *Plasmodium* species, in which a single isolate can differentiate and produce fertile male and female gametes that undergo sexual reproduction. In *Trypanosoma*, diploids may fuse to create an intermediate tetraploid that may undergo random chromosome loss through a parasexual cycle similar to *C. albicans* [47]. In *Toxoplasma gondii*, sexual outcrossing and subsequent self-mating generated highly virulent *T. gondii* clones that were responsible for a toxoplasmosis outbreak in Brazil and other global locales [48]. Unisexual reproduction preserved a well-adapted genomic configuration and generated abundant spores fueling this outbreak.

## Other Examples

Unisexual reproduction has emerged as an adaptive mechanism in eukaryotic microbes, but also extends beyond unicellular organisms. In plants, self-pollination is surprisingly common. Transitions from cross-pollination to self-pollination are frequently observed; for example, the model plant *Arabidopsis thaliana* reproduces almost exclusively through self-pollination (∼99%) yet retains the ability to outcross at a low frequency (∼1%). Such transitions are thought to enable provincial species with restricted niches to emerge as successfully dispersed cosmopolitan species. In *Bdelloid rotifers*, males, an extant sexual cycle, and meiosis all appear to be absent, and these organisms are only known to reproduce via parthenogenesis [49]. More than 80 unisexual species of fishes, insects, reptiles, and amphibians also reproduce via parthenogenesis. Facultative parthenogenesis, where sexual species resort to reproduction in the absence of a compatible partner, is more widespread than obligate parthenogenesis. In vertebrates and mammals, even facultative parthenogenesis is very rare due to genomic imprinting. However, some isolated female sharks have given birth to live young (all daughters) in the absence of a male partner [50]. Facultative selfing has been documented not only in sharks but also in komodo dragons [51], and some species of domesticated birds. Moreover, genetic manipulation of the imprinting mechanisms in female mice resulted in healthy, live offspring produced via parthenogenesis under laboratory conditions [52]. Both unisexual reproduction and parthenogenesis can mitigate some of the costs associated with sex, but a common consequence is a reduced level of genetic exchange or diversity.

## Conclusions

We now appreciate that eukaryotic microbial pathogens, including fungi and parasites, are not clonal and asexual, but rather have extant sexual cycles that are cryptic, parasexual, or even unisexual. Two of the three most common systemic human fungal pathogens (*Candida* and *Cryptococcus*) have retained extant sexual and parasexual cycles involving both bisexual and unisexual reproduction, which may provide a broader range of adaptive evolutionary strategies. Similar paradigms have now emerged for several eukaryotic parasites, suggesting this may be a general mode of adaptation for microbial pathogens, enabling them to preserve well-adapted genotypes. Given that sex is ubiquitous throughout the eukaryotic tree of life and yet we are confronted with a panoply of diverse mechanisms via which mating type (or sex) is specified and mating partners (or gametes) are distinguished and recognized, one possibility is that unisexual reproduction was the original ancestral form of sexual reproduction to which mating types and sexes were added later. If so, the finding that extant unisexual reproduction occurs in both fungal and parasitic pathogens may reflect a return to a more ancestral mode of reproduction rather than the emergence of an entirely new process promoting genetic change and exchange.
